# Primary vs. Secondary Antibody Deficiency: Clinical Features and Infection Outcomes of Immunoglobulin Replacement

**DOI:** 10.1371/journal.pone.0100324

**Published:** 2014-06-27

**Authors:** Sai S. Duraisingham, Matthew Buckland, John Dempster, Lorena Lorenzo, Sofia Grigoriadou, Hilary J. Longhurst

**Affiliations:** Immunology Department, Barts Health NHS Trust, London, United Kingdom; University of Thessaly, Faculty of Medicine, Greece

## Abstract

Secondary antibody deficiency can occur as a result of haematological malignancies or certain medications, but not much is known about the clinical and immunological features of this group of patients as a whole. Here we describe a cohort of 167 patients with primary or secondary antibody deficiencies on immunoglobulin (Ig)-replacement treatment. The demographics, causes of immunodeficiency, diagnostic delay, clinical and laboratory features, and infection frequency were analysed retrospectively. Chemotherapy for B cell lymphoma and the use of Rituximab, corticosteroids or immunosuppressive medications were the most common causes of secondary antibody deficiency in this cohort. There was no difference in diagnostic delay or bronchiectasis between primary and secondary antibody deficiency patients, and both groups experienced disorders associated with immune dysregulation. Secondary antibody deficiency patients had similar baseline levels of serum IgG, but higher IgM and IgA, and a higher frequency of switched memory B cells than primary antibody deficiency patients. Serious and non-serious infections before and after Ig-replacement were also compared in both groups. Although secondary antibody deficiency patients had more serious infections before initiation of Ig-replacement, treatment resulted in a significant reduction of serious and non-serious infections in both primary and secondary antibody deficiency patients. Patients with secondary antibody deficiency experience similar delays in diagnosis as primary antibody deficiency patients and can also benefit from immunoglobulin-replacement treatment.

## Introduction

Antibody deficiencies are defined by a loss of immunoglobulins or failure of immunoglobulin function, resulting in increased susceptibility to infection. In primary deficiencies inherited or sporadic genetic mutation(s), in some cases with unknown environmental cofactors, are suspected with no other known cause [1], [2]. Secondary antibody deficiency as a consequence of other diseases or medications can also occur [3]–[5]. Studies describe secondary antibody deficiencies as a result of haematological malignancy [6], [7], immunosuppressive [8]–[10] or anti-convulsant medications [11], protein-losing enteropathy [12], nephrotic syndrome and trauma [13]. Antibody deficiencies are associated with infections, immune dysfunction, end organ damage and significant morbidity and mortality [14], [15]. Immunoglobulin (Ig)-replacement for primary antibody deficiency is known to reduce infections, morbidity and mortality [16]–[18]. A small number of studies have demonstrated that (Ig)-replacement therapy is also effective in reducing severe infections in those with secondary antibody deficiency as a result of a haematological malignancy [19]–[22]. However as a whole, secondary antibody deficiencies are poorly described in the literature and clinical management guidance is usually extrapolated from experience with primary antibody deficiencies.

Although primary immunodeficiencies are rare, the advent of international registries has enabled more data to be pooled to further advance the understanding of clinical characteristics and treatment [23], [24]. By comparison, little has been published as yet about the overall prevalence of secondary antibody deficiencies, whether there is a delay in diagnosis and what the outcomes of Ig-replacement treatment are. The natural history of this heterogeneous group is not well understood, nor are we able to reliably identify who and when to treat. Since much information already published is on primary deficiencies, it may also be helpful to put secondary antibody deficiencies into context, relative to primary immunodeficiencies.

This study aimed to describe and compare features of primary and secondary antibody deficiency patients. We describe the characteristics of the cohort in terms of diagnosis, delay in diagnosis, bronchiectasis, possible causes of secondary immunodeficiency, concomitant disorders and immunological parameters. Serious and non-serious infection outcomes after Ig-replacement treatment are also compared in primary and secondary antibody deficiency patients.

## Patients and Methods

### Ethics Statement

All data was collected after obtaining written informed consent and in accordance with approval by the City and East London Research Ethics Committee.

### Study population and data collection

Adult subjects receiving Ig-replacement treatment in May 2013 seen in the immunodeficiency clinic at Barts Health NHS Trust were included in the study. Diagnoses were made by use of standard criteria where appropriate [25], [26]. The diagnosis of common variable immune deficiency (CVID) was made according to the criteria of decreased serum IgG, IgA and/or IgM, poor antibody response to vaccination and the exclusion of other causes of deficiency [27]. An ‘inflammatory’ CVID diagnosis was made based on a combination of clinical features, including persistent lymphadenopathy, (hepato)splenomegaly, synovitis, CT features of nodules or pulmonary infiltrates, cytopaenias, abnormal liver function in the absence of infection or other cause, or evidence of inflammatory infiltrates or granulomata on biopsy, in the absence of infectious or other causes. ‘Probable CVID’ subjects were those that fulfilled most CVID criteria but secondary causes could not be definitively excluded. Subjects with hypogammaglobulinaemia were defined as having serum IgG of <5.5 g/L, the lower limit of normal for our local laboratory, with or without low IgA or IgM. Specific antibody deficiency was defined by poor or absent antibody response after polysaccharide pneumococcal (Pneumovax-23) vaccination. Poor response was defined as achieving antibody levels of <0.35 mg/l, conferring basic protection against infection, to fewer than 8 of 13 serotypes; a more rigorous definition of antibody deficiency than that recommended by the American Academy of Allergy and Clinical Immunology [28]. Subclass deficiency subjects had low levels of one or more IgG subclasses in the context of normal total immunoglobulin isotypes [26], and were commenced on Ig-replacement treatment if symptomatic despite prophylactic antibiotics. Agammaglobulinaemia was characterised by panhypogammaglobulinaemia and infection onset <5 years of age – 11 subjects had X-linked agammaglobulinaemia (XLA) with a *BTK* mutation and one had autosomal recessive agammaglobulinaemia. The other primary immunodeficiencies were defined by genetic testing and/or clinical symptoms according to standard criteria [25]. The complete patient information dataset is available as the ‘Supplementary Dataset’ File.

Categorisation into ‘Primary’ or ‘Secondary’ antibody deficiency groups was based largely on patients fulfilling positive diagnostic criteria (e.g. CVID, XLA) for the primary group. For diagnosis of secondary antibody deficiency, clinical judgement took into account the timing of potential causes of deficiency (e.g. chemotherapy) and the development of symptoms or abnormal immunoglobulin levels. The ‘probable’ primary and secondary groups included those in whom a primary or secondary deficiency was suspected based on clinical history, but where other causes could not be excluded or where definitive information about timing of symptoms was not available (for example, more frequent/serious infections following immunosuppressive medication, suggesting a secondary deficiency). Demographic and diagnostic data were obtained retrospectively by reviewing medical records.

Diagnostic delay was defined as the time between patient-reported symptom onset or first documented serious infection, and antibody deficiency diagnosis. Subjects for whom dates of symptoms or diagnosis were unclear were excluded from this analysis. Serum IgG, IgA and IgM levels were recorded and the frequency of switched memory B cells (CD19^+^CD27^+^IgM^−^IgD^−^) determined as a percentage of total B cells using standard methods [29]. Analysis of serum immunoglobulin levels excluded those patients with specific/subclass deficiencies and was available for 58 primary group subjects and 27 secondary group subjects prior to Ig-replacement. Mean trough IgG levels over the year 2012/2013 were calculated - for patients starting Ig-replacement in the year 2012/2013, only IgG levels after the first 5 infusions were included to eliminate a loading effect.

### Immunoglobulin-replacement therapy

Patients were treated initially with a dose of 0.1 g/kg/week as recommended [26], [30] with dose adjustment to minimise infection frequency on an individual patient basis [16].

### Infection outcomes

For infection outcomes, data was collected for the year preceding initiation of Ig-replacement and for the year 01/06/12 to 31/05/13. For those where post-treatment infection data of <1 year was available (22 subjects), the number of infections were normalised on a pro-rata basis. Infections that overlapped with diagnosis and initiation of Ig-replacement were only included in the pre-treatment count. Infection data was available for a minimum of 53/126 primary and 28/39 secondary subjects pre-treatment, and for 115/126 primary and 37/39 secondary subjects post-treatment. ‘Serious infections’ were defined as infections requiring hospitalisation and/or intravenous antibiotics. ‘Non-serious’ infections included any mild or moderate infection, with or without antibiotic treatment. The number and type of non-serious infections were largely patient-reported with microbiological confirmation when appropriate and serious infection data was collected from medical records. For all historical pre-treatment data, infection counts were taken from clinical notes.

### Statistical methods

Data between primary and secondary groups were analysed by a two-tailed unpaired t-test with Welch's correction (unequal variance t-test). A two-tailed paired t-test was used for analysis of infection frequency pre- and post-treatment. Where comparisons were made between diagnostic sub-groups the Kruskal-Wallis test was used. Data on the number of switched memory B cells and bronchiectasis was only available for a small number of patients and was analysed using the Mann-Whitney test. P values of <0.05 were considered significant; all data analysis was carried out using GraphPad Prism 5.0.

## Results

### Immunodeficiency cohort

A total of 167 patients receiving Ig-replacement were identified (98 women and 69 men) including 113 with primary immunodeficiencies, 13 with a probable primary deficiency, 26 with secondary immunodeficiencies, 13 with a probable secondary immunodeficiency and 2 that could not be definitively classified. The subjects with secondary or probable secondary deficiencies were older (median age 64.5 yrs and 58 yrs) than the primary groups (45 yrs and 52 yrs). The most common diagnosis in the primary group was CVID accounting for 69.9% of the group (of which 20.3% had inflammatory CVID), and 10.6% of the primary group had agammaglobulinemia. A diagnosis of hypogammaglobulinemia was more common in the secondary and probable secondary groups (80.8% and 69.2%) and a smaller proportion had a specific or subclass defect (Table 1). The ‘definite’ and ‘probable’ groups had similar proportions of subjects with each type of diagnosis (Figure S1), thus for much of the further analysis the groups were combined and compared as primary (n = 126) and secondary (n = 39) groups.

**Table 1:** Immunodeficiency cohort on Ig-replacement treatment.

	PRIMARY	PROBABLE PRIMARY	SECONDARY	PROBABLE SECONDARY	UNKNOWN
**TOTAL**	**113**	**13**	**26**	**13**	**2**
Median age (range)	45 (17–91)	52 (30–81)	64.5 (40–82)	58 (28–79)	75.5 (74–77)
Male	47	5	10	5	1
Female	66	8	16	8	1
**CVID**	**79 (69.9%)**	**79 (69.9%)**	**-**	**-**	**-**
Non-inflammatory CVID	63	-	-	-	-
Inflammatory CVID	16	-	-	-	-
Probable CVID	-	8	-	-	-
**HYPOGAMMAGLOBULINAEMIA**	**5 (4.4%)**	**3 (23.1%)**	**21 (80.8%)**	**9 (69.2%)**	**2 (100%)**
**SPECIFIC OR SUBCLASS DEFICIENCY**	**10 (8.9%)**	**2 (15.4%)**	**4 (15.4%)**	**4 (30.8%)**	**-**
**AGAMMAGLOBULINAEMIA**	**12 (10.6%)**	**-**	**-**	**-**	**-**
XLA	11	-	-	-	-
Autosomal agammaglobulinaemia	1	-	-	-	-
**OTHER**	**7 (6.2%)**	**-**	**1 (3.8%)**	**-**	**-**
Combined deficiency	1	-	1	-	-
Good syndrome	1	-	-	-	-
ALPS	1	-	-	-	-
Ataxia telangiectasia	1	-	-	-	-
HyperIgE syndrome	1	-	-	-	-
WHIM syndrome	2	-	-	-	-

CVID indicates common variable immune deficiency; ALPS, autoimmune lymphoproliferative syndrome; and WHIM, warts hypogammaglobulinaemia infections and myelokathexis syndrome.

### Characteristics of secondary antibody deficiency

Based on the clinical history and/or timing of drug treatments relative to onset of symptoms, the most likely cause of secondary antibody deficiency in each subject was identified (Table 2). The most common likely cause was previous cancer chemotherapy for B cell lymphoma (11 subjects), of which most regimens included Rituximab (RTX). Four of these subjects had additionally undergone allogeneic stem cell transplantation. Although antibody deficiency can occur either as a result of the malignancy itself or secondary to treatment of the malignancy, based on the timing of symptoms, the antibody deficiency only manifested after chemotherapy in these B cell lymphoma patients. Immunosuppressive therapy for autoimmune or rheumatic diseases (5 subjects) and high dose or frequent corticosteroids for asthma or COPD (6 subjects) were also common likely causes. Three subjects had untreated B- or plasma cell clonal proliferations, and one had chronic hepatitis C. In the probable secondary deficiency group, the most common likely causes were corticosteroid, immunosuppressive or anti-convulsant medication use. No patient had protein loss such as nephrotic syndrome or protein-losing enteropathy as the cause of their severe antibody deficiency.

**Table 2:** Likely cause of secondary antibody deficiency in each subject.

	SECONDARY	PROBABLE SECONDARY
**UNTREATED MALIGNANCY**	**3**	**-**
CLL	1	-
MM	1	-
MGUS	1	-
**CHEMOTHERAPY**	**11**	**1**
with RTX	9	1
with stem cell transplant	4	-
**CORTICOSTEROIDS FOR ASTHMA OR COPD**	**6**	**5**
**AUTOIMMUNE OR RHEUMATIC**	**5**	**3**
RA or SLE	2	2
Wegener's granulomatosis	1	-
RA/SLE with RTX	1	1
SLE/Sjogren's with RTX	1	-
**OTHER**	**1**	**4**
Hepatitis C infection	1	-
Previous anti-convulsant drugs	-	3
Previous immunosuppressive drugs	-	1

CLL indicates chronic lymphocytic leukaemia; MM, multiple myeloma; MGUS, monoclonal gammopathy of unknown significance; RTX, Rituximab; RA, rheumatoid arthritis; and SLE, systemic lupus erythematosus.

The type of medications used before antibody deficiency diagnosis and the time between first use and diagnosis are shown for the secondary group (Table 3) and for individual patients that had received immunosuppressive medications (Table S1 in Tables S1). The most common medication used was corticosteroids (including that given as part of a chemotherapy regimen), followed by chemotherapy and RTX. The time between first use and diagnosis was shortest for immunosuppressive medications (median 0.5 yrs), followed by chemotherapy (2 yrs), Rituximab (3.5 yrs) and corticosteroids (5 yrs). For those that received more than one treatment of RTX (e.g. for recurrent lymphoma, or autoimmune disease) or more than one immunosuppressive medication before diagnosis, there was a longer time from first use to symptoms (3.5 yrs vs. 5 yrs for Rituximab and 0.5 yrs vs. 1.3 yrs for immunosuppressive drugs). This is likely due to the antibody deficiency only developing or becoming symptomatic after the second or subsequent treatment, suggesting a cumulative effect. Note that the timing between medication use and diagnosis was difficult to determine for most of the probable secondary group due to unclear clinical history data (this was usually the reason for classification as ‘probable’).

**Table 3:** Number of immunosuppressive therapies used by each group before symptom onset.

	SECONDARY		PROBABLE SECONDARY
	Number	Median years between first use and symptoms (range)	Number
**CORTICOSTEROIDS**	**18**	**5 (1–19)**	**10**
**CHEMOTHERAPY**	**11**	**2 (1–15)**	**1**
**RITUXIMAB**	**11**	**3.5 (0.5–10)**	**2**
More than one RTX treatment (including maintenance therapy)	6	5 (1.5–10)	1
**AT LEAST ONE OTHER IMMUNOSUPPRESSIVE DRUG**	**8**	**0.5 (0.5–5)**	**4**
**More than one other immunosuppressive drug**	**6**	**1.3 (0.5–4)**	**3**
Mycophenolate mofetil	3	-	2
Methotrexate	3	-	1
Cyclosporine	3	-	1
Cyclophosphamide	2	-	0
Hydroxychloroquinine	2	-	2
Leflunomide	1	-	1
**ANTI-CONVULSANTS**	**-**	**-**	**3**

Number indicates the number of therapies used (a single subject may have had more than one therapy).

### Diagnosis of antibody deficiency

**Figure 1:**
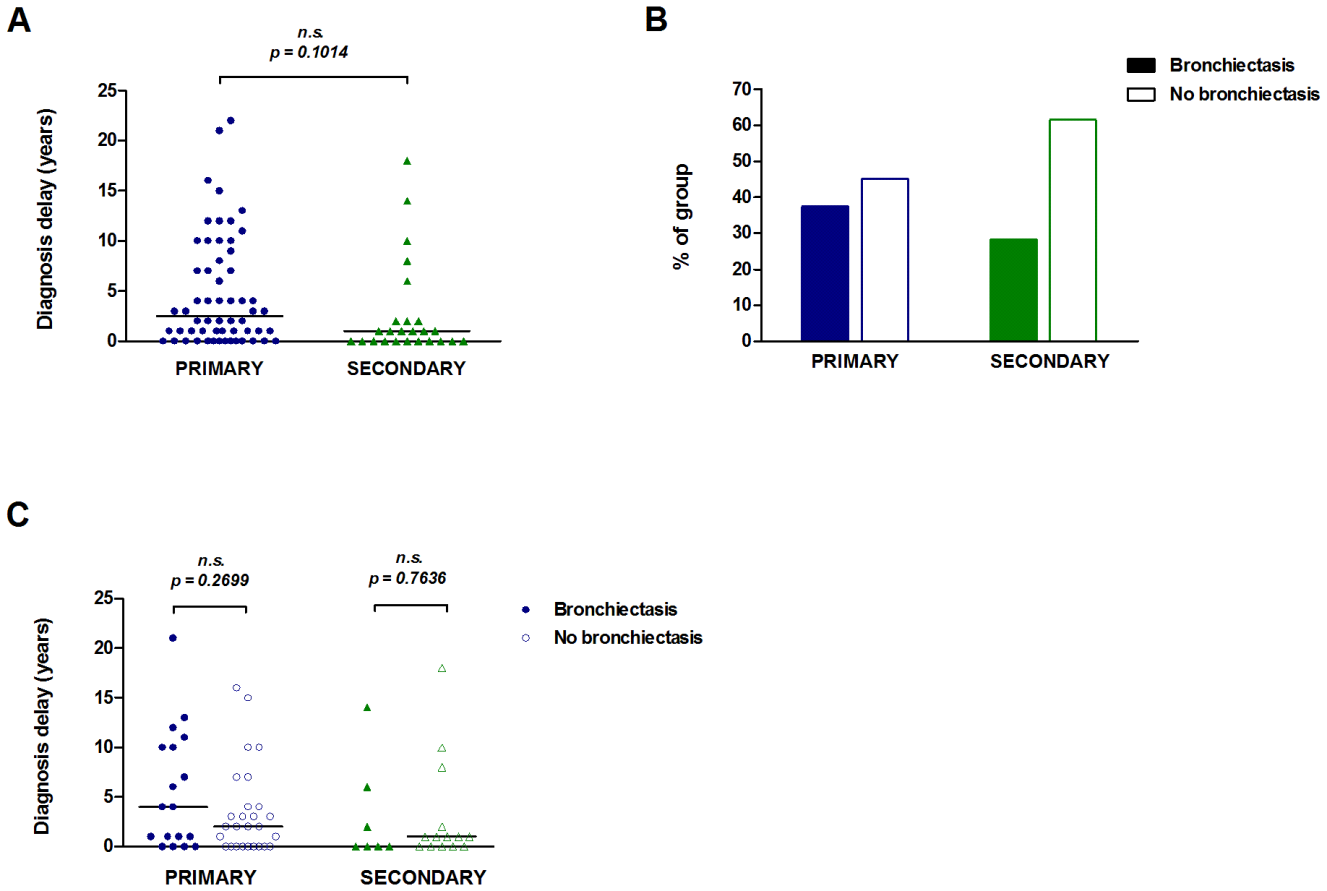
Diagnostic delay and the presence of bronchiectasis. Diagnostic delay (time between symptom onset and antibody deficiency diagnosis) was determined for the primary (n = 58) and secondary (n = 25) groups (A). The percentage of subjects with or without bronchiectasis (determined by high-resolution CT scan) is shown for each group (B). Diagnostic delay by bronchiectasis presence or absence is shown for the primary (n = 45) and secondary (n = 21) groups (C). The bars in panels A and C represent median values. Data in panel A were analysed by a two-tailed unequal variance t-test and data in panel C were analysed by a two-tailed Mann-Whitney test; n.s. non-significant (p values <0.05 were considered significant).

Delay in diagnosis of antibody deficiencies has been associated with greater infection-related morbidity [31], [32]. We assessed the diagnosis delay, defined as the number of years between symptom onset and diagnosis of antibody deficiency. There was no significant difference in diagnosis delay between the primary (median 2.5 yrs) and secondary (1 yr) groups (Figure 1A). In the primary group, the diagnosis of specific or subclass deficiencies was more delayed (median 11 yrs) than either CVID (2 yrs) or hypogammaglobulinemia (1 yr), although the number of subjects analysed was limited (Figure S2A). Similarly in the secondary group, diagnosis of antibody deficiency due to corticosteroid use (median 2 yrs) may be delayed in comparison to the chemotherapy (0.5 yrs) or malignancy (0.5 yrs) groups (Figure S2B).

Bronchiectasis is a complication of lung infections and has been shown to be associated with delay in diagnosis of antibody deficiency in some CVID cohorts [14], although not in others [15]. Bronchiectasis was identified in 37.3% of the primary group and 28.2% of the secondary group (Figure 1B). In the primary group bronchiectasis was most prevalent in the ‘Other immunodeficiencies’ and agammaglobulinaemia subjects and in ∼30% of CVID subjects (Figure S3A). In the secondary group half of the autoimmune/rheumatic sub-group and a third of the chemotherapy sub-group had bronchiectasis (Figure S3B). Despite this surprisingly high frequency of subjects with bronchiectasis, there was no significant difference in diagnostic delay between subjects with bronchiectasis and those without, for either primary or secondary antibody deficiency groups (Figure 1C).

Autoimmune disorders, suggesting immune dysregulation, were present in both primary and secondary immunodeficiency subjects (Table S2 in Tables S1), despite differences in the causes of antibody deficiency. The secondary group had a higher frequency of lymphoma in our cohort, although the incidence of Non-Hodgkins lymphoma is also known to be increased in older CVID patients [33]. In the primary group autoimmune disorders or lymphoma were likely to be manifestations of the primary deficiency whereas in the secondary group, antibody deficiency was a consequence of therapy for these disorders. Interestingly, there was a similarly high frequency of non-bronchiectatic chronic lung disease (asthma and/or COPD) in both groups (23.9% primary and 34.1% secondary), which may have implications for susceptibility to respiratory infections.

### Immunological parameters before Ig-replacement

**Figure 2:**
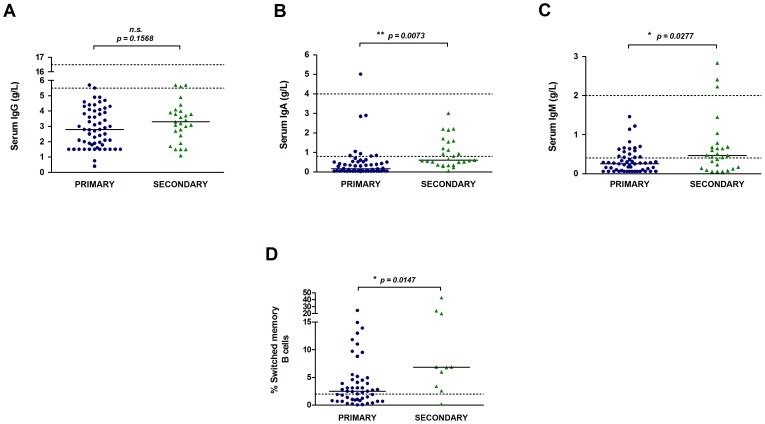
Immunological parameters before Ig-replacement treatment. Serum IgG (A), IgA (B) and IgM (C) levels in the year before Ig-replacement are shown for the primary (n = 58) and secondary groups (n = 27). Each symbol represents the mean value over the year for one subject and the bars represent the group median. The frequency of switched memory B cells (CD19^+^CD27^+^IgD^−^IgM^−^) as a proportion of peripheral blood B cells is shown for the primary (n = 50) and secondary (n = 10) groups (D). Dotted lines indicate the normal reference ranges for each. Data in panels A–C were analysed by a two-tailed unequal variance t-test and data in panel D were analysed by a two-tailed Mann-Whitney test; * p<0.05, ** p<0.01; n.s. non-significant.

Mean serum immunoglobulin levels were calculated for the year preceding initiation of Ig-replacement (excluding specific or subclass deficiency subjects). There was no significant difference in the pre-treatment IgG level between primary and secondary groups (median 2.79 g/L vs. 3.30 g/L) (Figure 2A). However, serum IgA (median 0.17 g/L vs. 0.60 g/L) and IgM (median 0.26 g/L vs. 0.47 g/L) levels were significantly higher in the secondary group (Figure 2B–C). Low frequencies of switched memory B cells (CD19^+^CD27^+^IgM^−^IgD^−^) have been associated with inflammatory/granulomatous and splenomegaly CVID clinical phenotypes [34], but the status of this B cell subset in those with secondary antibody deficiencies is unknown. Only one subject analysed in the secondary group had low numbers of switched memory B cells (<2%) and as a group, secondary antibody deficiency patients had significantly higher frequencies (median 6.9%) than the primary group (2.5%) (Figure 2D). The majority of patients previously treated with RTX had recovered normal circulating B cell numbers by the time of starting Ig-replacement, with the exception of one subject, and the two subjects on RTX maintenance treatment. Overall, those with secondary deficiency have similarly low IgG levels, although not as a result of low switched memory B cell frequencies.

### Infection outcomes after Ig-replacement treatment

The number of infections in subjects was compared before Ig-replacement (in the year preceding treatment) and after treatment (in the year 2012/2013). In this cohort, the primary group had been on Ig-replacement for a significantly longer time than the secondary group (median 3 yrs vs. 1 yr). The majority of subjects were on home therapy and a large proportion on sub-cutaneous administration (Table 4). Although the dose of IgG given (g/kg/4-weeks) was higher in the primary group, median trough IgG levels in the year 2012/2013 were similar for the primary (median 10.30 g/L) and secondary groups (9.75 g/L).

**Table 4:** Ig-replacement therapy in primary and secondary antibody deficiency patients.

	PRIMARY	SECONDARY	*p value*
**NUMBER OF SUBJECTS ON PROPHYLACTIC ANTIBIOTICS**			
Before starting Ig-replacement	47 (37.3%)	27 (69.2%)	
After starting Ig-replacement	64 (50.8%)	23 (60.0%)	
**MEDIAN IgG DOSE, g/week (RANGE)**	**11.20 (3.2–32.0)**	**10.10 (3.2–30.0)**	**n.s. (0.4645)**
(mean ± 95% C.I.)	(12.20±0.92)	(11.70±1.64)	
**MEDIAN IgG DOSE, g/kg (RANGE)**	**0.30 (0.10–1.30)**	**0.20 (0.10–2.00)**	**n.s. (0.1808)**
(mean ± 95% C.I.)	(0.41±0.91)	(0.36±0.22)	
**MEDIAN IgG DOSE, g/kg/4-weeks (RANGE)**	**0.70 (0.35–2.03)**	**0.53 (0.35–1.03)**	* 0.0110
(mean ± 95% C.I.)	(0.80±0.12)	(0.59±0.10)	
**MEDIAN TROUGH IgG, g/L (RANGE)**	**10.30 (5.46–18.93)**	**9.75 (6.35–13.70)**	***n.s. (0.1705)***
(mean ± 95% C.I.)	(10.62±0.50)	(9.92±0.63)	
**MEDIAN NUMBER OF YEARS ON Ig-REPLACEMENT (RANGE)**	**3 (<1–28)**	**1 (<1–9)**	* 0.0184
(mean ± 95% C.I.)	(3.77±0.87)	(2.54±0.74)	
**ROUTE OF ADMINISTRATION**			
IV	60 (47.6%)	13 (33.3%)	
SC	66 (52.4%)	26 (66.6%)	
**PLACE OF ADMINISTRATION**			
Home	80 (63.5%)	25 (64.1%)	
Barts Health	26 (20.6%)	9 (23.1%)	
Other local hospital	20 (15.9%)	5 (12.8%)	

Data were analysed with a two-tailed Mann-Whitney test; n.s., non-significant. IV indicates intravenous; and SC, sub-cutaneous.

**Figure 3:**
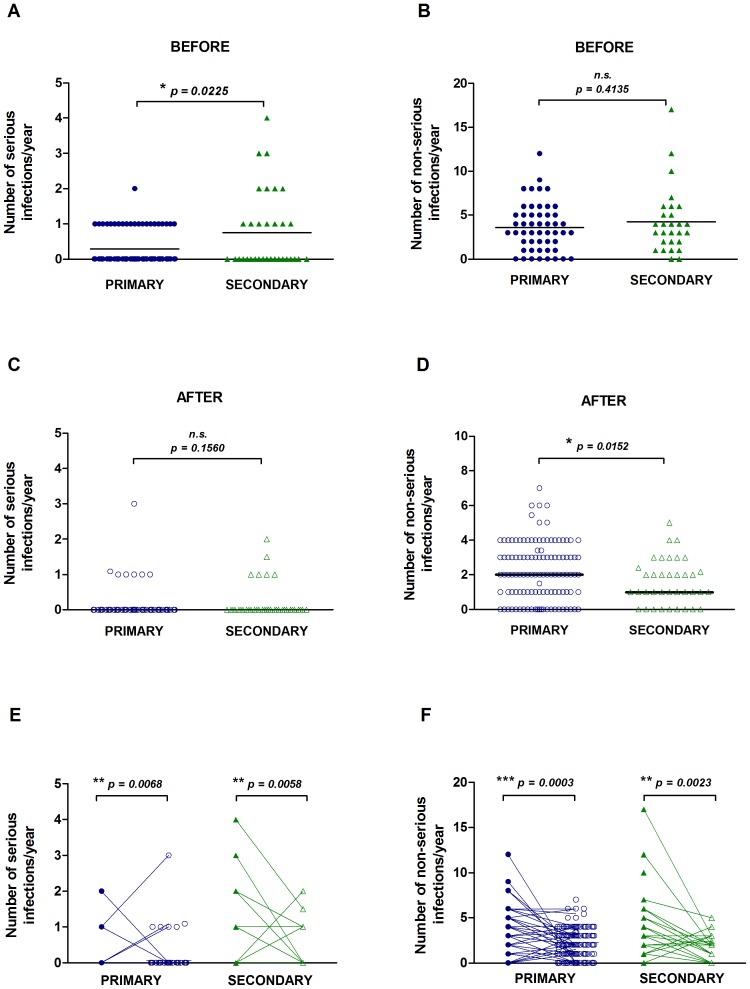
Number of serious and non-serious infections before and after Ig-replacement treatment. The number of serious infections requiring hospitalisation or IV antibiotics and the number of patient-reported non-serious infections in the year preceding Ig-replacement treatment (A–B) and in the year 2012/2013 (C–D) is shown. The bars represent the group medians. Serious (E) and non-serious infections (F) are shown for each patient before (filled symbols) and after (open symbols) treatment. Data in panels A–D were analysed by a two-tailed unequal variance t-test and data in panels E–F were analysed by a two-tailed paired t-test; * p<0.05, ** p<0.01, *** p<0.001; n.s. non-significant.

In the year before Ig-replacement, the secondary group had a significantly higher frequency of serious infections requiring hospitalisation and/or IV antibiotics, and a greater proportion of subjects with more than one serious infection compared to the primary group (Figure 3A). There was no significant difference in serious infections between secondary sub-groups such as chemotherapy vs. immunosuppressive (data not shown), suggesting that infection susceptibility was not related to the type of underlying disease. The number of non-serious infections (any patient-reported infections) before Ig-replacement did not differ between the primary and secondary groups (Figure 3B). The number of days on antibiotics before Ig-replacement also did not differ between the groups (data not shown). After Ig-replacement treatment, there was no significant difference in the number of serious infections between the primary and secondary immunodeficiency groups, but the number of non-serious infections was significantly lower in the secondary group (Figure 3C–D). Comparing infections in individual subjects before and after Ig-replacement, there was a significant decrease in serious and non-serious infections in both the primary and secondary groups (Figure 3E–F). The types of infections experienced are shown in Table S3 in Tables S1 - the most common serious infection before treatment was pneumonia and the most common non-serious infections were respiratory infections. The primary group tended to have more skin, sinus and ear infections than the secondary group. After treatment, there were an increased proportion of subjects that were infection-free in both the primary and secondary groups. The secondary group had a greater proportion of infection-free subjects than the primary group (23.1% vs. 16.6%) after Ig-replacement treatment.

The majority of secondary antibody deficiency patients received a trial of antibiotic prophylaxis as standard before Ig-replacement treatment [35]. There was a small increase in the proportion of subjects on antibiotic prophylaxis after Ig-replacement in the primary group and a small decrease in the secondary group (Table 4). Non-serious infections were more frequent in those on prophylactic antibiotics in the primary group before and after Ig-replacement (Figure S4A–B) which is probably due to antibiotic prophylaxis being used more often in those experiencing frequent infections.

## Discussion

We show that for our cohort, whatever the underlying cause of antibody deficiency, infection frequency is reduced by Ig-replacement. Secondary antibody deficiency patients respond at least as well, and possibly better, to antibody replacement, as evidenced by the similar reduction in serious infection frequency, greater reduction in non-serious infection frequency and lower immunoglobulin dose required by the secondary antibody deficiency group.

Registry studies have identified a smaller proportion (<1%) of antibody deficiency subjects with a secondary cause [36] than in our cohort. This could reflect exclusion of secondary antibody deficiencies from these registries, that patients with secondary antibody deficiency are more likely to be managed by specialists other than immunologists, or lack of recognition of the significance of secondary antibody deficiency. Most of the secondary antibody deficiency group (∼75%) had hypogammaglobulinaemia with a smaller proportion having a specific or subclass defect. In our cohort, the most common likely causes of secondary immunodeficiency were previous chemotherapy or immunosuppressive therapy, corticosteroids for chronic lung disease and less commonly haematological malignancies. Patients such as immunosuppressed solid organ transplant recipients who are also at risk of having secondary antibody deficiency [37] were not seen at our centre, neither were patients with low antibodies secondary to protein loss. This could reflect referral bias, but could equally reflect the increased susceptibility to infection of those with defects in immunoglobulin production and especially in those with the combination of B cell (pre)malignancy and immunosuppressive therapies, particularly therapies that target B cells.

In the secondary group, a delay of several years was noted between initial immunosuppressive therapy and the onset of infections. This delay was different for each drug type which may be due to the frequency, potency and regimens of the drugs used. Hypogammaglobulinaemia has previously been noted in RTX-treated patients after a delay of 1–2 years [38]. The longest delay was observed for corticosteroid use which may reflect intermittent use over a long period of time. Additionally, we observed a potentially longer time to symptom development after repeated treatments with one agent (e.g. RTX) or several agents (different immunosuppressive drugs). This may be indicative of a cumulative and combinatorial dose effect, which has been previously reported for repeated RTX cycles [39] and for cyclophosphamide followed by RTX [40]. However, studies of larger populations are needed to confirm this observation.

Since this study only includes those that are symptomatic and have been started on Ig-replacement, it is not clear what proportion of all those on such immunosuppressive drugs develop hypogammaglobulinaemia and how many become symptomatic. Estimates vary, with studies describing the incidence of hypogammaglobulinaemia as 14–35% following RTX treatment [9], [39]–[43], 27–54% for those with chronic lymphocytic leukaemia [6], [7], 17% of asthmatics on corticosteroids [44] and 40–63% of solid organ transplant recipients [45]. However not all with hypogammaglobulinaemia develop overt infections severe enough to require Ig-replacement, and those with low anti-pneumococcal antibodies may be particularly susceptible [7].

Despite the differences in causes of antibody deficiencies between the primary and secondary groups, they had a similar frequency of disorders related to immune dysregulation (infections, autoimmunity, malignancy) and for chronic lung disease. This overlap has led to diagnostic uncertainty which we have reflected in our patients classified as ‘probable’; the similar characteristics of the ‘probable’ and ‘definite’ groups in terms of diagnosis type, immune parameters and infection outcomes (data not shown), lead us to believe that the classification is reliable.

There was a surprisingly high incidence of bronchiectasis in our cohort (∼34%) compared to 11.2% in another study [46]. However, the ESID registry-based study found a Europe-wide incidence of 23%, which was noted to be higher in British cohorts [36]. Interestingly, up to half of those with secondary deficiency caused by chemotherapy or immunosuppressive drugs also had bronchiectasis. Although this was not associated with a diagnostic delay or poorer infection outcomes in our cohort (data not shown), bronchiectasis has been associated with reduced survival [15], implying that monitoring of end organ complications is important for secondary deficiency patients too. The higher incidence of bronchiectasis (and other complications) could explain the relatively high immunoglobulin dose and trough levels for our cohort [24]. Although data was limited, there did not appear to be any overall difference in diagnostic delay between the primary and secondary groups in our cohort. Greater diagnostic delay may occur for those on intermittent corticosteroids: recurrent respiratory infections might erroneously be attributed to chronic lung disease rather than underlying immune deficiency.

The primary antibody deficient patients, of whom the majority had CVID or XLA, had lower baseline levels of serum IgM and IgA and fewer switched memory B cells than the secondary patients before Ig-replacement therapy. Sub-groups of CVID patients have been identified based on the number of total, switched memory and transitional B cells [47], [48]. Low frequencies of switched memory B cells in particular have been associated with splenomegaly and granulomatous/inflammatory CVID [34]. Differences in B cell subset numbers could yield clues as to the mechanism of antibody deficiency. Low total B cell numbers may be a result of a defect in early B cell differentiation and low switched memory B cells may reflect a germinal centre defect [34], [49]. The molecular mechanisms of secondary antibody deficiencies are virtually unknown and are likely to be heterogeneous depending on the cause of deficiency. The normal number of switched memory B cells observed in our cohort of secondary antibody patients may suggest a post-germinal centre (GC) defect in antibody production, such as a defect in plasmablast differentiation or poor survival and/or replenishment of the plasma cell pool. Patients with low levels of immunoglobulins before RTX or immunosuppressive treatments are more likely to develop hypogammaglobulinaemia [9], [37], suggesting that some people may have a pre-existing predisposition to antibody deficiency. Treatment with RTX targets CD20^+^ B cells, including the pro-B-cell to mature GC phenotypes, plasma cells are unaffected, and follicular dendritic and T-helper cell numbers in tissue may also be reduced [50]. Future work to study this compartment in detail prior to RTX may help identify the at-risk phenotype.

In the year preceding Ig-replacement, the secondary group had a greater number of serious infections and several patients had more than one. Since there did not appear to be any diagnostic delay between the primary and secondary groups, secondary deficiency patients may be relatively asymptomatic until they develop a serious infection that warrants referral to an immunologist. The greater occurrence of serious infections may also be explained by the older age and co-morbidities, including drug and disease-associated immune defects of the secondary group. An alternative explanation is that only those secondary antibody deficient patients with severe enough symptoms to be on Ig-replacement were included, whereas Ig-replacement is initiated as first-line treatment for most confirmed primary antibody deficiency patients. The rate of decline of immunoglobulin levels in some secondary immunodeficiency patients may also have a bearing on the rate of emergence of infectious complications.

After Ig-replacement, infections were significantly reduced in both groups thus the cause of antibody deficiency may not be important in itself. Ig-replacement has been previously shown to be effective in reducing infections in haematological malignancy patients with hypogammaglobulinaemia [19], [20], [22] but has not previously been studied in patients selected according to antibody deficiency rather than underlying cause. Trough IgG levels were similar in both groups and did not correlate with infections (data not shown) due to our practice of increasing dosage if too many breakthrough infections occur [16]. Additionally, in our cohort the primary group had been on Ig-replacement for a longer time than the secondary group (3 yrs vs. 1 yr) which may mean that their trough IgG levels had already been optimised to minimise breakthrough infections, making the lower post-treatment infection rate in the secondary group even more noteworthy. The dose of IgG used, which appears to be effective, was lower in the secondary group despite similar pre-treatment levels, suggesting reduced peripheral consumption or that replacement itself may have a greater effect on endogenous Ig production.

This study is the first to describe a cohort of unselected secondary antibody deficiency compared to primary antibody deficiency patients and to compare outcomes on Ig-replacement therapy. Secondary antibody deficiency manifesting as hypogammaglobulinaemia or specific/subclass deficiencies can occur with haematological malignancies or after cell depletion therapy, chemotherapy, immunosuppressive or corticosteroid medications. Despite having a greater number of serious infections in the year before Ig-replacement therapy, secondary antibody deficiency patients had significantly fewer non-serious infections than the primary group after therapy. Therefore, regardless of whether the cause of symptomatic antibody deficiency is primary or secondary, both groups benefit from Ig-replacement therapy.

## Supporting Information

Figure S1 **Primary and secondary antibody deficiency diagnoses.** The number of subjects with each diagnosis classified as having a primary, probable primary, secondary, probable secondary or unknown antibody deficiency is shown.
(TIF)

Figure S2 **Diagnostic delay by primary and secondary antibody deficiency sub-groups.** Diagnostic delay (time between symptom onset and antibody deficiency diagnosis) was analysed by diagnosis for the primary group (A) and by likely cause of deficiency for the secondary group (B). Data were analysed by the Kruskal-Wallis test, p values of <0.05 were considered significant; n.s. non-significant.
(TIF)

Figure S3 **Bronchiectasis by primary and secondary antibody deficiency sub-groups.** The percentage of each sub-group with or without bronchiectasis (determined by high-resolution CT scan) is shown by diagnosis for the primary group (A) and by likely cause of deficiency for the secondary group (B).
(TIF)

Figure S4 **Prophylactic antibiotics and the occurrence of non-serious infections.** The number of non-serious infections in those receiving antibiotic prophylaxis compared to those not on prophylactic antibiotics before (A) and after (B) Ig-replacement is shown. Data were analysed by a two-tailed unequal variance t-test; * p<0.05.
(TIF)

Tables S1 Contains the following files: Table S1. Immunosuppressive therapies used by individual patients before diagnosis. Table S2. Disorders in primary and secondary antibody deficiency patients. Table S3. Number and type of infections experienced by the primary and secondary group before and after Ig-replacement.
(DOCX)

Dataset S1 **A file of the full dataset is provided.**
(XLS)
