# Supplementary material for: Primary vs. Secondary Antibody Deficiency: Clinical Features and Infection Outcomes of Immunoglobulin Replacement
Source: PLoS One. 2014 Jun 27;9(6):e100324. doi: 10.1371/journal.pone.0100324 (PMC4074074; doi:10.1371/journal.pone.0100324)
Supplement: Tables S1 — Contains the following files: Table S1. Immunosuppressive therapies used by individual patients before diagnosis. Table S2. Disorders in primary and secondary antibody deficiency patients. Table S3. Number and type of infections experienced by the primary and secondary group before and after Ig-replacement. (DOCX) [file pone.0100324.s005.docx]

**Table S1. Immunosuppressive therapies used by individual patients before diagnosis.**

| **Patient** | **Indication for immunosuppressive therapy** | **Medications, number of cycles (dates)** | **Date of diagnosis** |
| --- | --- | --- | --- |
| 128 | Autoimmune – SLE/Sjogrens | Corticosteroids (?- ), MMF (2005-), AZA (2005-), RTX 4 cycles (2007) | ? |
| 129 | Lymphoblastic lymphoma | Chemotherapy [unknown (2003)], cyclophosphamide (2004) | 2005 |
| 131 | Mantle cell lymphoma, stem cell transplant, GvHD | Chemotherapy [CVAD (2008)], RTX 3 cycles (2008), MMF (2009), Cyclosporine (2009) | 2012 |
| 134 | Follicular lymphoma | Chemotherapy [chlorambucil (2004), fludarabine (2005), RTX-CHOP 6 cycles (2006)] | 2006 |
| 135 | Myelodysplasia treatment, stem cell transplant, GvHD | Chemotherapy [ICE (2006)], corticosteroids (2006-2007), cyclosporine (2006-2007), RTX 4 cycles (2008) | 2012 |
| 136 | Follicular lymphoma | Chemotherapy [chlorambucil, FMD (1992-2006), RTX-CHOP 6 cycles (2002)] | 2010 |
| 137 | Follicular lymphoma | Chemotherapy [fludarabine, CHOP (2000), chlorambucil (2002), bortezomib (2002), RTX 4 cycles (2001), RTX-ICE 3 cycles (2006)] | 2006 |
| 138 | Follicular lymphoma | Chemotherapy [chlorambucil (1992, 1994), FMD (1996), RTX (1998, 1999)] | 1999 |
| 139 | Autoimmune - RA | Corticosteroids (2009-), MTX (2011-) | 2011 |
| 140 | Autoimmune – RA/SLE | Corticosteroids (?-), MMF (?-?), MTX (?-?), RTX 8 cycles (2006-2011), AZA (2009-2011) | 2012 |
| 144 | MZ lymphoma | Chemotherapy [unknown (2003), chlorambucil (2008)] | 2012 |
| 145 | Autoimmune - RA | Corticosteroids (2005-), MTX (2009), leflunomide (2010) | 2010 |
| 148 | Autoimmune – Wegener’s granulomatosis | Corticosteroids (?-), cyclophosphamide (2009), AZA (2010) | 2010 |
| 149 | Follicular lymphoma | Chemotherapy [chlorambucil (2010), RTX 4 cycles (2010), RTX 3 cycles (2011)] | 2012 |
| 150 | MZ lymphoma | Chemotherapy [chlorambucil (1995), fludarabine (1995, 1999, 2009), FMD (2001)], RTX unknown cycles (2000), RTX 5 cycles (2009) | 2010 |
| 151 | Non-Hodgkins lymphoma stem cell transplant | Chemotherapy [CHOP (2000), ESHAP (2001)], RTX unknown cycles (2001) | ? |

Dates are shown where known. MMF indicates mycophenolate mofetil; MTX, methotrexate; AZA, azathioprine; RA, rheumatoid arthritis; SLE, systemic lupus erythematosus; FMD, fludarabine, mitoxantrone, dexamethasone; RTX, Rituximab; CHOP, cyclophosphamide, adriamycin, vincristine, prednisolone; GvHD, graft-versus-host disease; ICE, ifosfamide, carboplatin, etoposide; DHAP, dexamethasone, cytarabine, cisplatin; ESHAP, etoposide, methylprednisolone, cytarabine, cisplatin.

**Table S2. Disorders in primary and secondary antibody deficiency patients.**

|  | **PRIMARY** | **SECONDARY** |
| --- | --- | --- |
| **NONE (INFECTIONS ONLY)** | **56 subjects** | **2 subjects** |
| **TOTAL NUMBER OF NON-INFECTIOUS DISORDERS** | **74** | **44** |
| **AUTOIMMUNE** | **21 (28.4%)** | **9 (20.5%)** |
| ITP/AIHA | 7 | 1 |
| Solid organ | 4 | - |
| Connective tissue | 1 | 7 |
| Other | 2 | - |
| Arthropathy | 7 | 1 |
| **CANCER** | **5 (6.8%)** | **17 (38.6%)** |
| Lymphoma | 3 | 13 |
| ALL/CLL/MM/MGUS | 1 | 3 |
| Other | 1 | 1 |
| **GASTROINTESTINAL (GI) INFLAMMATORY** | **6 (8.1%)** | **1 (2.3%)** |
| **INFLAMMATORY CVID** | **16 (21.6%)** | **-** |
| Lung | 11 | - |
| Liver | 2 | - |
| Spleen | 3 | - |
| **SPLENECTOMY** | **5 (6.8%)** | **2 (4.5%)** |
| **CHRONIC LUNG DISEASE** | **21 (28.4%)** | **15 (34.1%)** |
| COPD | 8 | 5 |
| Asthma | 13 | 10 |

Percentages shown are as a proportion of total disorders for each group (a single subject may have had more than one disorder). ITP indicates immune thrombocytopaenic purpura; AIHA, autoimmune haemolytic anaemia; COPD, chronic obstructive pulmonary disease; ALL, acute lymphoblastic leukaemia; CLL, chronic lymphocytic leukaemia; MM, multiple myeloma; and MGUS, monoclonal gammopathy of unknown significance.

**Table S3. Number and type of infections experienced by the primary and secondary group before and after Ig-replacement.**

|  | **PRIMARY** | | **SECONDARY** | |
| --- | --- | --- | --- | --- |
|  | **BEFORE** | **AFTER** | **BEFORE** | **AFTER** |
| **SERIOUS** | **22** | **8** | **25** | **7** |
| Pneumonia | 14 (63.6%) | 1 (12.5%) | 13 (52.0%) | 0 (0.0%) |
| Sepsis | 2 (9.1%) | 1 (12.5%) | 8 (32.0%) | 3 (42.9 %) |
| Meningitis | 3 (13.6%) | 0 (0.0%) | 0 (0.0%) | 0 (0.0%) |
| Infective exacerbation of asthma or COPD | 1 (4.5%) | 3 (37.5%) | 1 (4.0%) | 2 (28.6%) |
| Other | 2 (9.1%) | 3 (37.5%) | 3 (12%) | 2 (28.6%) |
| **NON-SERIOUS** | **211** | **269** | **122** | **56** |
| Respiratory | 125 (59.2%) | 176 (65.4%) | 95 (77.9%) | 44 (75.9%) |
| UTI | 8 (3.8%) | 22 (8.2%) | 3 (2.5%) | 4 (6.9%) |
| Diarrhoea | 18 (8.5%) | 10 (3.7%) | 6 (4.9%) | 2 (3.4%) |
| Skin | 11 (5.2%) | 9 (3.3%) | 2 (1.6%) | 1 (1.7%) |
| Sinusitis | 19 (9.0%) | 34 (12.6) | 7 (5.7%) | 3 (5.2%) |
| Otitis | 13 (6.2%) | 3 (1.1%) | 6 (4.9%) | 0 (0.0%) |
| Conjunctivitis | 2 (0.9%) | 4 (1.5%) | 1 (0.8%) | 1 (1.7%) |
| HSV | 14 (6.6%) | 5 (1.9%) | 1 (0.8%) | 1 (1.7%) |
| Other | 1 (0.4%) | 6 (2.2%) | 1 (0.8%) | 0 (0.0%) |
| **INFECTION-FREE SUBJECTS** | **4 (3.2%)** | **21 (16.6%)** | **1 (2.6%)** | **9 (23.1%)** |

% indicates percentage of all serious or non-serious infections experienced by the group. COPD indicates chronic obstructive pulmonary disease; UTI, urinary tract infection; and HSV, herpes simplex virus.
